# Supplementary material for: Using a patient-reported outcome to improve detection of cognitive impairment and dementia: The patient version of the Quick Dementia Rating System (QDRS)
Source: PLoS One. 2020 Oct 15;15(10):e0240422. doi: 10.1371/journal.pone.0240422 (PMC7561106; doi:10.1371/journal.pone.0240422)
Supplement: S1 File — (PDF) [file pone.0240422.s001.pdf]

QUICK DEMENTIA RATING SYSTEM (QDRS)

1. The following descriptions characterize changes in the patient's cognitive and functional abilities. You are asked to compare the patient now to how they used to be – the key feature is ***change***.

2. Check **one answer** for each category that best fits the study participant.

NOTE - not all descriptions need to present to choose an answer.

1. MEMORY AND RECALL	
☐0	No obvious memory loss or slight inconsistent forgetfulness that does not interfere with everyday function
☐0.5	Consistent mild forgetfulness or partial recollection of events that may interfere with performing everyday activities; repeats questions/statements, misplaces items, forgets appointments
☐1	Mild to moderate memory loss; more noticeable for recent events; interferes with performing everyday activities
☐2	Moderate to severe memory loss; only highly learned information remembered; new information rapidly forgotten
☐3	Severe memory loss, almost impossible to recall new information; long-term memory may be affected

2. ORIENTATION	
☐0	Fully oriented to person, place, and time nearly all the time
☐0.5	Slight difficulty keeping track of time; may forget day or date more frequently than in the past
☐1	Mild to moderate difficulty keeping track of time and sequence of events; forgets month or year; oriented to familiar places but gets confused outside of familiar areas; gets lost or wanders
☐2	Moderate to severe difficulty, usually disoriented to time and place (familiar and unfamiliar); frequently dwells in past
☐3	Only oriented to their name, although may recognize family members

3. DECISION MAKING AND PROBLEM SOLVING ABILITIES	
☐0	Solves everyday problems; handles personal business and financial affairs well; decision-making abilities consistent with past performance
☐0.5	Slight impairment or takes longer to solve problems; trouble with abstract concepts; decisions still sound
☐1	Moderate difficulty with handling problems and making decisions; defers many decisions to others; social judgment and behavior may be slightly impaired; loss of insight
☐2	Severely impaired in handling problems, making only simple personal decisions; social judgment and behavior often impaired; lacks insight
☐3	Unable to make decisions or solve problems; others make nearly all decisions for patient

4. ACTIVITIES OUTSIDE THE HOME	
☐0	Independent in function at usual level of performance in profession, shopping, community activities, religious services, volunteering or social groups
☐0.5	Slight impairment in these activities compared to previous performance; slight change in driving skills; still able to handle emergency situations
☐1	Unable to function independently but still may attend and be engaged; appears “normal” to others; notable changes in driving skills; concern about ability to handle emergency situations
☐2	No pretense of independent function outside the home; appears well enough to be taken to activities outside the family home but generally needs to be accompanied
☐3	No independent function or activities; appear too ill to be taken to activities outside the home

5. FUNCTION AT HOME AND HOBBY ACTIVITIES	
☐0	Chores at home, hobbies and personal interests are well maintained compared to past performance
☐0.5	Slight impairment or less interest in these activities; trouble operating appliances (particularly new purchases)
☐1	Mild but definite impairment in home and hobby function; more difficult chores or tasks abandoned; more complicated hobbies and interests given up
☐2	Only simple chores preserved, very restricted interest in hobbies which are poorly maintained
☐3	No meaningful function in household chores or with prior hobbies

6. TOILETING AND PERSONAL HYGEINE	
☐0	Fully capable of self-care (dressing, grooming, washing, bathing, toileting)
☐0.5	Slight changes in abilities and attention to these activities
☐1	Needs prompting to complete these activities but may still complete independently
☐2	Requires some assistance in dressing, hygiene, keeping of personal items; occasionally incontinent
☐3	Requires significant help with personal care and hygiene; frequent incontinence

7. BEHAVIOR AND PERSONALITY CHANGES	
☐0	Socially appropriate behavior in public and private; no changes in personality
☐0.5	Questionable or very mild changes in behavior, personality, emotional control, appropriateness of choices
☐1	Mild changes in behavior or personality
☐2	Moderate behavior or personality changes, affects interactions with others; may be avoided by friends or distant family
☐3	Severe behavior or personality changes; making interactions with others unpleasant or avoided all together

8. LANGUAGE AND COMMUNICATION ABILITIES	
☐0	No language difficulty or occasional word searching; reads and writes as well as in past
☐0.5	Consistent mild word finding difficulties, using descriptive terms or takes longer to get point across, mild problems with comprehension, decreased conversation; may affect reading and writing
☐1	Moderate word finding difficulty in speech, cannot name objects, marked reduction in work production; reduced comprehension, conversation, writing and/or reading
☐2	Moderate to severe impairments in speech production or comprehension; has difficulty communicating thoughts to others; limited ability to read or write
☐3	Severe deficits in language and communication; little to no understandable speech

9. MOOD	
☐0	No changes in mood, interest or motivation level
☐0.5	Occasional sadness, depression, anxiety, nervousness or loss of interest/motivation
☐1	Daily mild issues with sadness, depression, anxiety, nervousness or loss of interest/motivation
☐2	Moderate issues with sadness, depression, anxiety, nervousness or loss of interest/motivation
☐3	Severe issues with sadness, depression, anxiety, nervousness or loss of interest/motivation

10. ATTENTION AND CONCENTRATION	
☐0	Normal attention, concentration and interaction with his/her environment and surroundings
☐0.5	Mild problems with attention, concentration, and interaction with environment and surroundings, may appear drowsy during day
☐1	Moderate problems with attention and concentration, may have staring spells or spend time with eyes closed, increased daytime sleepiness
☐2	Significant portion of the day is spent sleeping, not paying attention to environment, when having a conversation may say things that are illogical or not consistent with topic
☐3	Limited to no ability to pay attention to external environment or surroundings

Copyright 2013 *The Quick Dementia Rating Scale* James E. Galvin and New York University Langone Medical Center
