# Supplementary material for: Using a patient-reported outcome to improve detection of cognitive impairment and dementia: The patient version of the Quick Dementia Rating System (QDRS)
Source: PLoS One. 2020 Oct 15;15(10):e0240422. doi: 10.1371/journal.pone.0240422 (PMC7561106; doi:10.1371/journal.pone.0240422)
Supplement: Attachment — Submitted filename: PLoSOne_20200828.docx [file pone.0240422.s003.docx]

Abstract:

1. Please define the statistic “Ps.”

Introduction:

1. The authors provide a paragraph describing the limitations of performance measures and brief tests. They then state that PROs can overcome those limitations but do not explain how, for example, PROs could improve upon the potential for bias by education, language, and culture in performance measures.
2. Similarly, is there literature suggesting that patients at risk for cognitive impairment would be more willing to take a self-administered assessment for dementia at home vs. in office?

Methods:

1. Please provide the specific name of your center.
2. I’m confused about the study design. The authors state that the study population consisted of 269 patient-caregiver dyads to their center but that welcome packets with the QDRS were mailed prior to the office visit. How can the test be administered before determination of eligibility?
3. If welcome packets were mailed to additional patients, how many chose to respond? How did these patients differ from those included in the study in terms of demographics, diagnosis, and disease severity?
4. I suggest calling the “CDR” the “CDR Global Score” to describe the specific summary measure of the CDR.
5. Are all of the tests and assessments described in the section under the title “Caregiver ratings of patient cognition and behavior” provided by the caregiver and not the patient?

Results:

1. In Table 1, consider providing either in the table or the text, the possible ranges for each assessment of the assessments.
2. Given the small numbers in the tables, I don’t think the authors should provide a global p-value for race. Perhaps one for CDR 0 vs. CDR>0 would be more informative?
3. Is “Response Counts” showing the levels of the CDR Global Score? Please specify.
4. In the Reliability and Scale Score Feature of the Patient QDRS, the authors mention “random error.” I don’t think that’s an accurate description of the goal of analyzing internal consistency.
5. Tables 5 and 6 need more labels to descript coefficients, effects, 95% CIs, etc.
6. I’m not following why you would combine the QDRS with the MoCA if the goal is to have an instrument that can be self-administered. I would recommend cutting this section unless the authors can provide substantial motivation.
7. I think the ROC figures can be cut; providing the AUC is sufficient.
8. In Table 7, specify what statistics are in the cells. Mean (sd)?
9. Are the p-values in Table 7 adjusted for multiple comparisons? Why formally test instead of providing CIs?
